# Biomechanical evaluation of modified and traditional cortical bone trajectory technique on adjacent segment degeneration in transforaminal lumbar interbody fusion—finite element analysis

**DOI:** 10.1186/s12891-023-07103-4

**Published:** 2024-01-02

**Authors:** Abudusalamu Tuoheti, Yang Xiao, Yixi Wang, Abulikemu Maimaiti, Rui Zhang, Alafate Kahaer, Abuduaini Tuoheti, Xianghui Wu, Paerhati Rexiti

**Affiliations:** 1 Department of Spine Surgery, The First Affiliated Hospital of Xinjiang Medical University, Urumqi, China; 2 Xinjiang Medical University, Urumqi, China; 3 Beijing University of Posts and Telecommunications, Bei Jing, China; 4 Ministrv of Education, Key Laboratory of High Incidence Disease Research in Xingjiang (Xinjiang Medical University), Urumqi, China; 5 Xinjiang Clinical Research Center for Orthopedics, Urumqi, China

**Keywords:** Cortical bone trajectory, Transforaminal lumbar Interbody fusion, Adjacent segment degeneration, Finite element analysis

## Abstract

**Objectives:**

Modified cortical bone trajectory (MCBT) technique was proposed by our team in previous studies, but its biomechanical properties at adjacent segments have not been discussed yet. Therefore, the purpose of this study is to investigate the biomechanical properties of modified cortical bone trajectory (MCBT) technique on adjacent segment degeneration (ASD) in transforaminal intradiscal lumbar disc fusion (TLIF) compare to traditional bone trajectory (TT) technique and cortical bone trajectory (CBT) technique.

**Methods:**

The four human cadaveric lumbar specimens were provided by the anatomy teaching and research department of Xinjiang Medical University and four intact finite element models of the L1-S1 segment were generated. For each of these, three transforaminal lumbar interbody fusion procedures with three different fixation techniques were reconstructed at the L4-L5 segment, as follows: TT-TT (TT at both L4 and L5 segments), CBT-CBT (CBT at both L4 and L5 segments), MCBT-MCBT (MCBT at both L4 and L5 segments). The range of motion and von Mises stress of the intervertebral disc of the L3-L4 and L5-S1 segments were recorded with a 400N compressive load and 7.5 Nm moments in flexion, extension, left–right bending, and left–right rotation.

**Results:**

The peak ROM of the L3-L4 segment in the MCBT-MCBT group was reduced by 10.5%, 6.1%, 12.2%, 4.1%, and 1.5% in flexion, extension, left–right bending, and left rotation compared to the TT-TT group and reduced by 1.8%, 5.5%, 10.0%, 12.8%, and 8.8% in flexion, left–right bending, and left–right rotation compared to the CBT-CBT group, respectively. The MCBT-MCBT group has the lowest peak ROM of the L3-L4 segment in flexion, left bending, and right rotation, the lowest peak ROM of the L5-S1 segment in extension and right rotation, and the lowest peak von Mises stress of the intervertebral disc at the L5-S1 segment in right rotation compared to the TT-TT and CBT-CBT group. In addition, the peak von Mises stress at the L3-L4 segment was lowest and more dispersed in all motions, the MCBT-MCBT group exhibited lower peak ROM of the L5-S1 segment in flexion, extension, and right rotation, and showed lower peak von Mises stress of the disc at the L5-S1 segment in flexion, extension, and right rotation compared with the TT-TT group.

**Conclusion:**

The modified cortical bone trajectory technique may have a beneficial effect on reducing the incidence of ASD in the L4-L5 TLIF model compared to the traditional bone trajectory technique and cortical bone trajectory technique.

## Introduction

Adjacent segment degeneration (ASD) was first reported by Anderson et al. in 1956 [1], is a pathological process that produces abnormalities in the adjacent segments after lumbar fusion. It has been found that ASD is a chronic disease, and Paul Park et al. [2] proposed that age, fusion fixation, small joint injury in adjacent segments, number of fused segments, gender, degree of osteoporosis, body mass index, and surgical methods may be important factors in the degeneration of adjacent segments.

German surgeon Harms first introduced transforaminal lumbar interbody fusion (TLIF) in 1986 [3] and is an intervertebral fusion technique developed based on posterior lumbar interbody fusion (PLIF). TLIF can significantly reduce the risk of dural sac rupture and nerve injury, and this advantage has been well-proven in clinical practice [4]. Traditional bone trajectory (TT) technique became the "gold standard" in spinal fixation techniques [5]. Most operators currently apply the TT fixation technique to TLIF surgery. However, it is worth mentioning that recent studies have shown that the TT technique has deficiency points such as the tendency to screw loosen after surgery, especially in elderly patients with osteoporosis [6, 7]. Umehara et al. [8] reported that the reduction or loss of the anterior convexity angle of the lumbar spine after internal fixation fusion, or even the appearance of kyphosis and coronal instability, as well as three-dimensional instability of the spine, are important causes of ASD from the internal fixation system after fusion surgery. Therefore, the above shortcoming of the TT technique may develop as a long-term potential factor for accelerating the development of ASD in TLIF.

Santoni et al. [9] proposed the cortical bone trajectory (CBT) technique in 2009. Different from the TT technique, the CBT technique has more contact with the cortical bone increasing the pull-out strength by 30% [9] and the insertion torque by 1.7 times [10]. However, we found that the CBT technique did not fully utilize the cortical bone at the medial border of the pedicle and lateral margin of the superior endplate of the vertebral body. Therefore, we proposed the modified cortical bone trajectory (MCBT) technique since the year 2018 [11–13]. Fujiwara et al. [14] found that CBT screw insertion torque was positively correlated with screw length, negatively correlated with the distance from the screw to the medial border of the pedicle, and negatively correlated with the distance from the screw to the superior endplate. This is also consistent with the MCBT concept of having a longer cortical screw tract and closer placement to the medial and inferior walls of the pedicle, as well as closer placement of the screw head to the lateral aspect of the vertebral body's superior endplates, which improves the biomechanical properties of the screws by allowing the screw tract to come into contact with more cortical bone [12, 13]. In a previous study, our team evaluate the biomechanics effect of the modified cortical bone trajectory (MCBT) technique with other traditional internal fixation systems by comparing the von Mises stress, displacement, von Mises stress on L4–L5 segment of the lumbar spine using numerical simulation method, and demonstrated that the MCBT technique showing better biomechanical characteristics in the fixed segment compared to the other fixation techniques [15]. However, the biomechanical properties of the MCBT-MCBT technique at adjacent segments in the TLIF FE model have not been discussed previously. In this study, the biomechanical properties of the MCBT-MCBT technique at adjacent segments were investigated and compared with the TT-TT and CBT-CBT techniques.

## Material and methods

### Model development and validation

Construction and validation of the intact FE models were constructed and validated in the previous study [16]. Material properties were set in line with the previous models [16]. The finite element (FE) models comprised 5 lumbar vertebrae with the sacrum, 7 ligaments, and 5 intervertebral discs including cranial and caudal endplates. Each segment incorporated facet joint cartilages with an initial space of 0.5 mm [17]. Cortical bone and endplate thicknesses were defined as 0.5–1 mm[17] and 1 mm [18], respectively. The nucleus pulposus simulates a fluid, incompressible substance that occupies 44% of the disk volume [19]. The trihedral elements were adopted for each selected elements. Informed consent was obtained as previously reported.

### Construction of surgical models

**Fig. 1:**
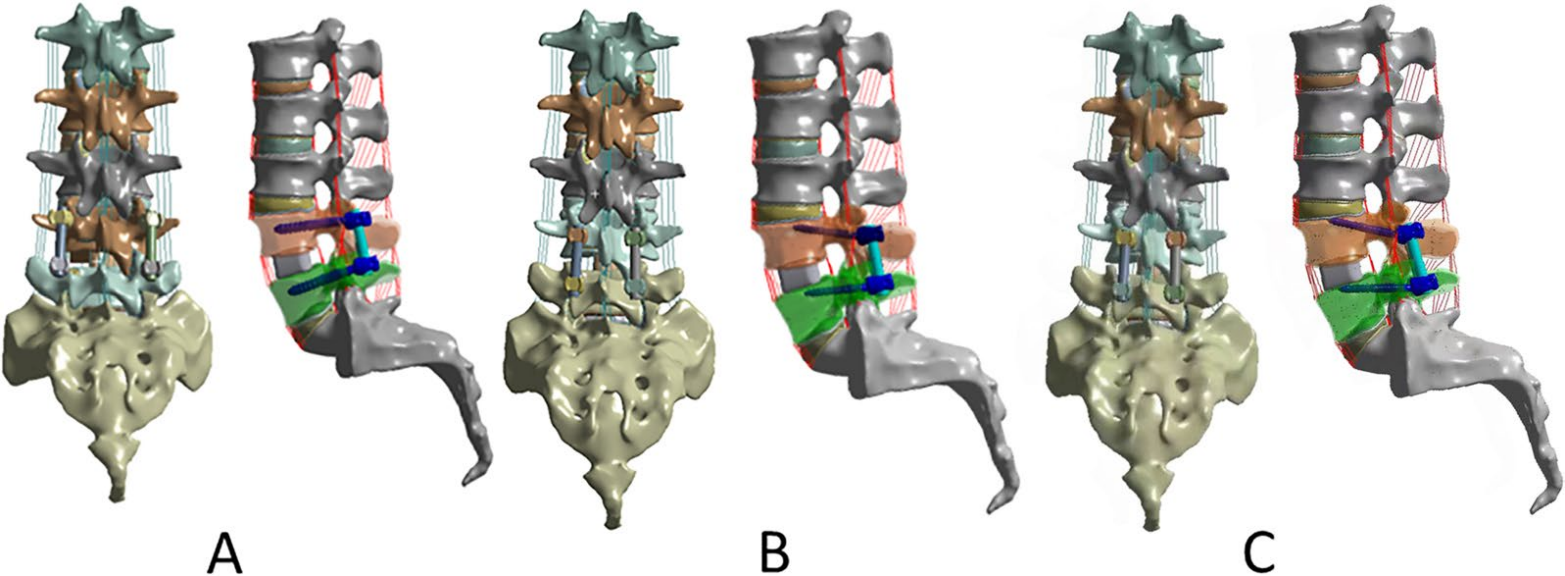
FE models of L1-S1 lumbar spine with TLIF at the L4-L5 segment with three different fixation techniques. **A** TT at L4 and L5 (TT-TT) [16]; **B** CBT at L4 and L5 (CBT-CBT) [16]; **C** MCBT at L4 and L5 (MCBT-MCBT) [20]

Three different posterior fixations were established: (1) TT-TT group, TT at both L4 and L5 segments (Fig. 1A) [16]; (2) CBT-CBT group, CBT at both L4 and L5 segments (Fig. 1B) [16];(3) MCBT-CBT group, MCBT at both L4 and L5 segments (Fig. 1C) [20]. For CBT screws the starting point of the screw is located at the intersection of the horizontal line 1 mm below the transverse process and the vertical line of the outer edge of the ipsilateral superior articular process with the left pedicle projecting in the 5 o'clock direction and the right pedicle projecting in the 7 o'clock direction, using the clock face for orientation [21]. For MCBT screws the entry point is located at the intersection of the horizontal line 1 mm below the transverse process and the medial wall tangent line of the pedicle which was shown in the previous studies [12]. The TT, CBT, and MCBT techniques have different trajectories and entry points for screw placement. The varying trajectories intrinsic to each technique dictate optimal screw lengths. After a review of related academic literature and analysis of relevant anatomical data [10, 22–25], we selected TT screws with the diameter in 6.0 mm and the length in 45 mm, CBT screws with the diameter in 5.0 mm and the length in 35 mm, MCBT screws with the diameter in 5.0 mm and the length in 40 mm. The accuracy of screw placement with the same trajectory was strictly controlled for all models.

### Boundary and loading conditions

In all of the models, we have successfully fixed and restrained the sacrum to prevent any movement at the sacral base and lower lumbar levels which ensures a stable base for applying loads and moments. Then, 400 N compressive load and 7.5 Nm torque were applied to the reference point on the superior surface of the L1 vertebra to simulate flexion, extension, left–right bending, and left–right rotation. The ROM and von Mises stress of the intervertebral disc at L3-L4 and L5-S1 segments were recorded and analyzed by using the ANSYS Workbench 19.1 (ANSYS, Inc., Canonsburg, PA, USA).

### Statistical analysis

SPSS 27.0 software was used for data analysis and process. The means of quantitative data were expressed as mean ± standard deviation, and we used paired t-test for the analysis of variance. When differences were statistically significant, post hoc tests were performed using the least significant difference (LSD) method. All results were considered statistically significant at P < 0.05.

## Results

### Model validation

In this finite element (FE) model, we have generated three distinct mesh resolutions (mesh 1, mesh 2, and mesh 3), and conducted a convergence analysis by comparing the percentage difference in von Mises stress between mesh 1 and mesh 3 and between mesh 2 and mesh 3. Between Mesh 1 and Mesh 3, predicted von Mises stress showed percentage differences of 4.06% in cortical bone, 1.45% in cancellous bone, 0.03% in cartilaginous endplate, 0.14% in nucleus pulposus, and 0.17% in annulus. These values were reduced to 2.48%, 0.55%, 0.03%, 0.02%, and 0.01% respectively when comparing Mesh 2 and Mesh 3. The results demonstrated that the differences in von Mises stress among the components were minimal between mesh 2 and mesh 3, thus validating the selection of mesh 2 as a highly reliable option [16]. Subsequently, we compared each segment's range of motion (ROM) after subjecting them to a compressive load of 400 N and moments of 7.5 Nm to simulate flexion, extension, left–right bending, and left–right rotation. The ROM of the intact model exhibited similarity with the findings and variational trends reported by Yamamoto et al. [26], Shim et al. [27], Huang et al. [28], and Lo et al. [29], thus validating the efficacy of the FE model for further biomechanical analysis [16].

### ROM of L3-L4 segment

The TT-TT group exhibited the highest peak ROM of the L3-L4 segment (1.51 ± 0.207°) in extension. Compared with the TT-TT group, the CBT-CBT group showed an 8.9%, 9.8%, and 7.1% reduction in the peak ROM of the L3-L4 segment in flexion, extension, and left bending. Right bending, left- right rotation increased by 6.1%, 11.4%, and 9.2%, respectively. Compared with the TT-TT group, the peak ROM of the L3-L4 segment in the MCBT-MCBT group decreased by 10.5%, 6.1%, 12.2%, 4.1%, and 1.5% in flexion, extension, left–right bending, and left rotation, respectively. And there was an increase of 0.4% in right rotation. In addition, compared with the CBT-CBT group, the peak ROM of the L3-L4 segment in the MCBT-MCBT group showed a 1.8%, 5.5%, 10.0%, 12.8%, and 8.8% decrease in flexion, left–right bending, left–right rotation, and a 3.9% increase in posterior extension, respectively.

### ROM of L5-S1segment

The figure shows that the TT-TT group showed the highest peak ROM (1.45 ± 0.349°) in extension. Compared with the TT-TT group, the CBT-CBT group showed 5.6%, 13.8%, 3.7%, 5.4%, and 3.7% decreases in the peak ROM of the L5-S1 segment in flexion, extension, left bending, and left–right rotation. And right bending increased by 12.4%. Compared with the TT-TT group, the peak ROM of the L5-S1 segment in the MCBT-MCBT group decreased by 2.5%, 15.8%, and 7% in flexion, extension, and right rotation. In contrast, there were increases of 8.9%, 15.5%, and 1% in left- right bending, and left rotation. Compared with the CBT-CBT group, the peak ROM of the L5-S1 segment in the MCBT-MCBT group decreased by 2.4% and 3.4% in extension and right rotation, respectively. And it increased by 3.4%, 12.3%, 3.6%, and 6.3% in flexion, left–right bending, and left rotation.

### Von Mises stress of intervertebral disc at L3-L4 segment

Compared with the TT-TT group, the CBT-CBT group showed a 1.76%, 0.05%, and 0.06% decrease in peak von Mises stress of the disc L3-L4 segment in right bending, and left–right rotation, respectively. And it increased by 0.20%, and 0.21% in flexion, and extension. In addition, compared with the TT-TT group, the peak von Mises stress of the L3-L4 segment in the MCBT-MCBT group decreased by 1.4% in right bending, while it increased by 0.33%, 0.90%, 0.39%, 0.47%, and 0.36% in flexion, extension, left bending, left–right rotation, respectively. Compared with the CBT-CBT group, peak von Mises stress of the L3-L4 segment in the MCBT-MCBT group increased by 0.13%, 0.69%, 0.38%, 0.33%, 0.53%, and 0.41% in flexion, extension, left–right bending, left–right rotation.

### Von Mises stress of intervertebral disc at L5-S1 segment

Compared with the TT-TT group, the CBT-CBT group showed a 2.66%, 2.86%, 8.94%, 9.77%, and 2.79% decrease in peak von Mises stress of the disc at L5-S1 in flexion, extension, left–right bending, and left rotation, except in right rotation which increased by 2.04%. Compared with the TT-TT group, peak von Mises stress of L5-S1 in the MCBT-MCBT group decreased by 0.39%, 0.75%, 3.33%, and 0.78% in flexion, extension, right bending, and right rotation. In contrast, it increased by 1.63% and 3.58% in left bending and left rotation. Compared with the CBT-CBT group, the peak von Mises stress of L5-S1 in the MCBT-MCBT group was reduced by 2.80% in right rotation. There was an increase of 2.27%, 2.13%, 10.44%, 6.65%, and 6.26% in flexion, extension, left–right bending, and left rotation, respectively.

## Discussion

Adjacent Segment Degeneration (ASD) has become one of the major complications after lumbar interbody fusion, and sometimes it may lead to further complications such as lumbar instability and lumbar spinal stenosis, which seriously affects the postoperative outcome of patients and causes more financial burden [30]. By following 912 patients with lumbar fusion at 5 and 10 years postoperatively, Sears et al. [31] found that the incidence of ASD increased year by year, it is 17% and 31%, respectively. The first step in TLIF is to implant an internal fixation screw to restrict the movement of the fused segment in all directions to enhance the anatomical stability of the three-dimensional structure of the spine, and then bone rongeur is used to remove unilateral inferior articular process and enter the spinal canal, decompression is performed at the lower lumbar recess of the internal fixation fusion segment to relieve nerve compression. Finally, bone fragments or appropriate cage are implanted to complete lumbar fusion [3], thus it will promoting the bony fusion between the fused vertebrae. The increased long-term load on the posterior column of the lumbar spine after fusion fixation increases the shear forces acting on the adjacent segments which in turn leads to changes in the biomechanics of the spine and accelerates the development of ASD. In addition, Lee et al. [32]further demonstrated that postoperative hyperactivity of adjacent segments and stress overload in adjacent discs are important factors in accelerating adjacent segment degeneration (ASD). How to try to avoid ASD during TLIF surgery is one thing that spine surgeons need to think seriously about. It has been shown that superior adjacent segment degeneration is particularly common after fusion [33], which may be caused by a shift of the spinal movement center to the proximal segment of the fusion segment after spinal fusion fixation, with the superior adjacent segment bearing more compensatory biomechanical effects [34]. However, the anatomical characteristics of the human body itself are that the anatomical structure of the lower lumbar spine is stronger than that of the upper lumbar spine, and the stability of the lower lumbar spine in all directions is stronger than that of the upper lumbar spine as a means of resisting the gravitational force to which the body is subjected [35, 36]. Therefore, it is unreasonable to assume more compensatory biomechanical effects on the upper lumbar spine, which are relatively weak in anatomical structure, after the spinal motion center is shifted upward by lumbar internal fixation fusion in clinical practice, so it is very important to equalize the stresses on the upper and lower adjacent segments intraoperatively, which can play a very important role in the postoperative outcome.

### Rom of the adjacent segment in three fixation techniques

In the present study, our data showed that the peak ROM of the adjacent segment in the TT-TT group was slightly higher than the CBT-CBT group under all motion states, and the peak von Mises stress of the disc at L3-L4 segment in the TT-TT and CBT-CBT group were essentially equal, while the peak von Mises stress of the disc at L5-S1 segment in the TT-TT group was higher than the CBT-CBT group, which is generally consistent with the results of data from Zhang R et al. [16] Whereas the results in the article by Zhang L et al. [37] showed that the peak ROM of the adjacent segments in the TT-TT group was smaller than the CBT-CBT group in all motion conditions, the peak von Mises stress of the disc at L3-L4 segment was essentially equal in the TT-TT and CBT-CBT group, while the peak von Mises stress of the disc L5-S1 segment in the TT-TT group was slightly smaller than the CBT-CBT group. The reason for this difference in data results was explained in our previous study [16], and this article focuses on the biomechanical properties of the MCBT-MCBT technique on the incidence of adjacent segments after TLIF surgery.

**Fig. 2:**
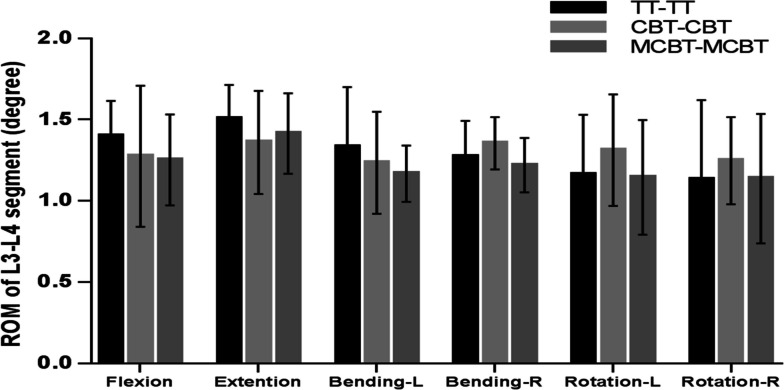
ROM of L3-L4 segment in three fixation techniques

**Fig. 3:**
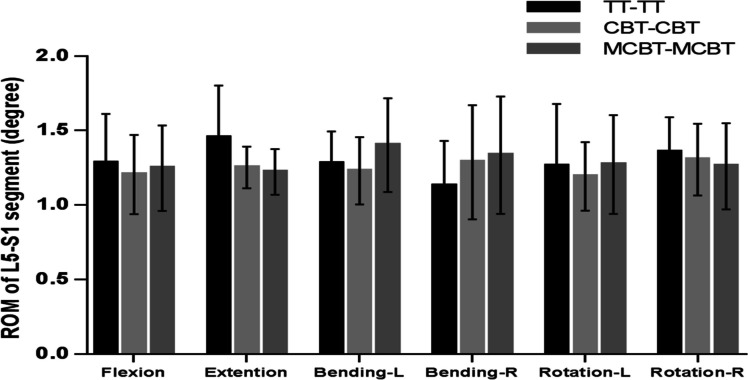
ROM of L5-S1 segment in three fixation techniques

In the peak ROM of upper adjacent segments (L3-L4), we found that the TT-TT group exhibited the highest peak ROM in extension (1.5075 ± 0.2071°), and the MCBT-MCBT group showed the lowest peak ROM in right rotation (1.1375 ± 0.39870°) and the highest peak ROM in extension (1.4150 ± 0. 24,772°). Some scholars have demonstrated that the higher ROM of adjacent segments after fusion causes a higher possibility of adjacent segment degeneration (ASD) [37]. Compared to the TT-TT group, the peak ROM of the L3-L4 segment in the MCBT-MCBT group was reduced by 10.5%, 6.1%, 12.2%, 4.1%, and 1.5% in flexion, extension, left–right bending, and left rotation. It was reduced by 1.8%, 5.5%, 10.0%, 12.8%, and 8.8% in flexion, left–right bend, and left–right rotation compared to the CBT-CBT group. The MCBT-MCBT group showed lower peak ROM of the upper adjacent segment (L3-L4) almost in all motions, except for a 0.4% increase in right rotation compared to the TT-TT group and a 3.9% increase in extension compared to the CBT-CBT group (Fig. 2). This general trend suggests that the MCBT technique reduces the compensatory biomechanical effect of the adjacent segment (L3-L4) which is more able to protect the relatively weak upper adjacent segment joint capsule (L3-L4), thus the long-term accumulation of this protective function has some effect in delaying the development of ASD. In the peak ROM of the lower adjacent segment (L5-S1), the MCBT-MCBT group showed lower peak ROM in flexion, extension, and right rotation, but higher peak ROM in left–right bending, and left rotation compared to the TT-TT group. Additionally, the MCBT-MCBT group showed lower peak ROM in extension and right rotation, but higher peak ROM in flexion, left–right bending, and left rotation compared with the CBT-CBT group (Fig. 3). There may be three reasons for this result, ① different screw sizes: TT screws (6.0 mm in diameter and 45 mm in length), CBT screws (5.0 mm in diameter and 35 mm in length), and MCBT screws (5.0 mm in diameter and 40 mm in length) ② Different screw placement points: The TT screw placement point is the intersection point between the vertical line of the outer edge of the upper articular process and the horizontal line of the midpoint of the transverse process; the CBT screw placement point is located at the lateral point of the articular isthmus; the MCBT screw point was placed at the incisal edge of the medial wall of the pedicle. ③ Different angle of screws: the cranio-cauda angle of the TT screw tract is 5° to 15°, and the mediolateral angle is 0° to 30°; the CBT screw tract has a cranio-cauda angle of 10° and the mediolateral angle of 25° [38]; the MCBT screw tract has cranio-cauda angle of 22° and the mediolateral angle of 20° to 30° [12]. The MCBT technique increases the thickness of the marginal cortex at the edge of the screw insertion point and increases the stability of the screw by contacting the medial wall of the pedicle and the lateral side of the upper endplate, especially increasing the effective screw length within the pedicle [12, 13]. Under the action of force conduction, more overload stress was concentrated in the fusion segment of the MCBT-MCBT group and the firmer lower adjacent segment (L5-S1), thus the center of rotation of spinal movement was not significantly shifted upward. This is conducive to showing the lower peak ROM of the L3-L4 segment which relatively weak anatomical structure, while the little higher peak ROM of the L5-S1 segment which more stable anatomical structure, which further suggests that the MCBT technique may tend to equalize the ROM of the in the upper and lower adjacent segments.

### Von Mises stress of intervertebral disc at the adjacent segment in three fixation techniques

The intervertebral disc and the two-facet joints constitute the three-joint complex, which maintains the stability of the lumbar spine and transfers load, guiding and restricting the movement of the lumbar spine. Lee and Langrana [34] used the L3-S1 segment of the cadaveric spine to study the stress changes of adjacent discs and demonstrated that the mechanical stress concentration of adjacent discs and joint surfaces occurred after lumbar fusion, which accelerated the incidence of ASD. It is proven that the greater the stress endured on the intervertebral discs, the higher the probability of adjacent segment degeneration [37].

**Fig. 4:**
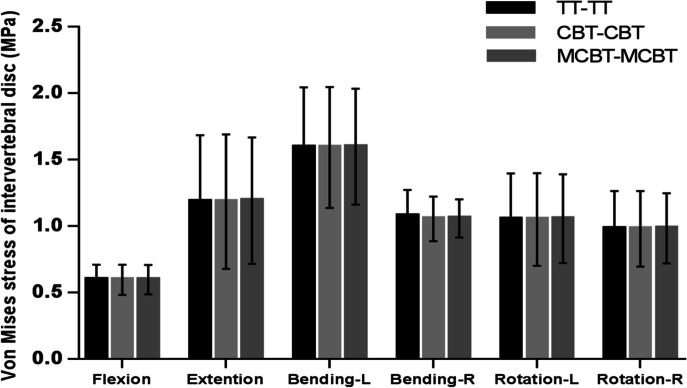
Von Mises stress of intervertebral disc at L3-L4 segment in three fixation techniques

**Fig. 5:**
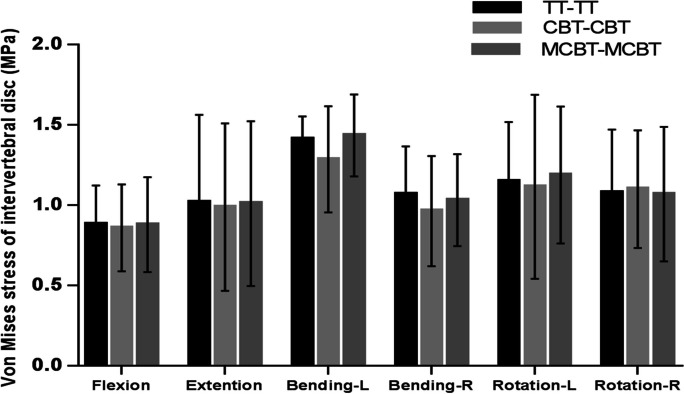
Von Mises stress of intervertebral disc at L5-S1 segment in three fixation techniques

**Fig. 6:**
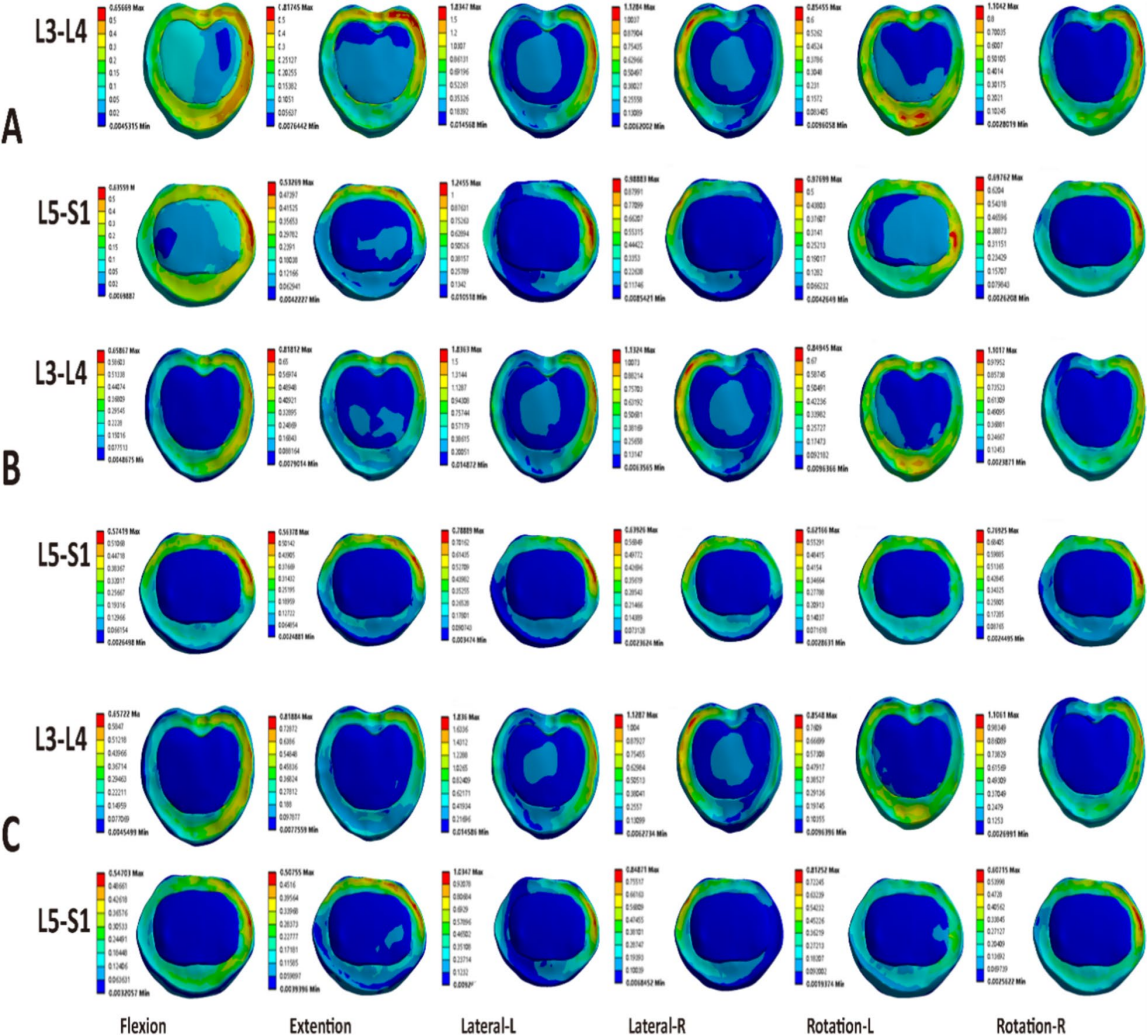
Stress programs over the screw in three fixation models. **A** TT-TT; **B** CBT-CBT; **C** MCBT-MCBT

In the present experiment, the disc stress at the L3-L4 segment was lowest and more dispersed in all motions in the TT-TT, CBT-CBT, and MCBT-MCBT groups (Fig. 6). The data showed that the three fixation techniques essentially showed the same stress on the upper adjacent disc after fusion. The MCBT-MCBT group showed the highest peak disc stress in left bending (1.5980 ± 0.43642 MPa) and the lowest peak disc stress in flexion (0.5971 ± 0. 11,060 MPa) at the L3-L4 segment (Figs. 4, 6). In von Mises stress of the disc at the L5-S1 segment, the MCBT-MCBT group exhibited the highest peak disc stress (1.4342 ± 0.25578 MPa) in left bending and the lowest peak disc stress (0.8779 ± 0.29620 MPa) in flexion. Compared with the TT-TT group, the MCBT-MCBT group showed a decrease of 0.39%, 0.75%, 3.33%, and 0.78% in anterior flexion, posterior extension, right bending, and right rotation, respectively. In contrast, there was an increase of 1.63% and 3.58% in left bending and left rotation. Compared to the CBT-CBT group, the MCBT-MCBT group showed higher peak von Mises stress of the disc at L5-S1segemnt in almost all motion conditions, with a reduction of 2.80% only in right rotation (Figs. 5, 6). The MCBT technique appeared to subject the inferior lumbar L5-S1 to a higher stress load. The three fixation techniques all increased the overload stress on the adjacent intervertebral discs to a certain extent, but the MCBT-MCBT group, which showed a higher peak stress of the L5-S1 intervertebral disc, may cause the degeneration of the L5-S1 intervertebral disc earlier. In the axial position, the angle of the L1-L2 facet joints of the lumbar spine is almost vertical, while the L5-S1 facet joints increase in width, become shallower, and tend to be coronal to prevent the spine from moving forward. Therefore, the direction of the articular angle of the lumbar facet changes from top to bottom and gradually from sagittal to coronal position. In addition, the articular capsule is more robust than that of the upper and lower lumbar vertebrae, with more attachment to the edge of the articular process (13 mm inside), and these anatomical structural changes further increase the stability of the lower lumbar vertebrae [35, 36]. Therefore, we believe that the above disadvantage of the MCBT-MCBT group is compensated by the robust anatomical characteristics of L5-S1 to some extent. The L5-S1 segment below the fixed segment (L4-L5) is more capable of carrying heavier stress loads due to its own anatomy compared to the L3-L4 segments above. Further cadaver research is still needed to better understand how the higher von Mises stress of the L5-S1 segment in MCBT-MCBT groups to patient outcomes. Furthermore, the MCBT technique allows the screw head end to reach the lateral edge of the horizontal column of the vertebral body. Even if the front end of the MCBT screw breaks through the upper endplate cortex of the vertebral body, due to the large external angle of the modified screw placement, only the peripheral part of the fibrous annulus of the intervertebral disc will be damaged, while the key part of the nucleus pulposus will not be damaged like CBT screw, thus the occurrence of adjacent vertebral disease (ASD) will be avoided [11–13].

### Facet joints violation of three fixation techniques

The facet joint is an important joint of the spine and forms a three-joint complex with the intervertebral disc. It is an important component of the lumbar motion segment and has great significance for the stability of the lumbar spine [39]. Symmetrical facet joints are subject to equal loads on both sides and have a high degree of interdependence with the intervertebral disc, where changes in one can affect the other two and vice versa [40]. Damage and degeneration of the proximal articular facet joints will further increase the pressure on the proximal intervertebral disc [41]. In lumbar fusion fixation, the choice of screw placement point, the patient's conditions, and the surgeon's experience may cause the injury of the facet joint. The injury of facet joints can cause relative displacement and angular deformity of the spine, resulting in spinal instability and postoperative lumbar and back pain. At the same time, the protrusion joints after injury are prone to dissociation, resulting in excessive disc torsion, thus also accelerating disc degeneration and increasing the incidence of postoperative ASD [42–45]. Chung et al. [46] and He et al. [47] reported that different locations of screw placement during surgery would impact the invasion of adjacent facet joints.

From the position of the screw placement point and facet joint, the TT screw placement point was at the intersection of the transverse horizontal line and pedicle midline, close to the facet joint (Fig. 1A). The intraoperative transverse process is not easy to reveal and has anatomic morphological variation, which requires extensive peeling and pulling of soft tissues such as fascia, muscle, and ligament to accurately determine the screw placement point location, which greatly increases the invasion rate of the lower facet joint of the upper adjacent segment (2.1% ~ 30.4%) [48]. Compared with TT screws, CBT screws were placed at the intersection of the horizontal line 1 mm below the transverse process and the vertical line of the outer edge of the lateral articular process, which was relatively far away from the articular process, and positioning could be completed by peeling off a small amount of soft tissue (Fig. 1B). The incidence of articular process injury was 0–11.8% [49]. Our previous studies have found that the CBT technique still has little shortcomings such as the tail of the screw being not enough far away from the facet joint, which is easy to impact and cause discomfort to patients. Rexiti P et al. [12, 13] set the screw placement point of the MCBT at the intersection point between the 1 mm horizontal line of the lower edge of the transverse process and the medial wall of the pedicle, also used the accessory spines of the lumbar spine near the base of the transverse process as an anatomical reference to solve the problem of difficult identification of the lower edge of the transverse process, to facilitate the positioning process of screw placement (Fig. 1C). Compared with CBT screws, the longitudinal axis of MCBT screws is moved inward, the screw placement point is closer to the midline level, and the placement point is as far away from the facet joint as possible, thus the screw tail can have more room for movement and can avoid impact with the facet joint, and the joint capsule is protected.

### Damage to adjacent muscles and soft tissues in three fixation techniques

It has been suggested that the extensive destruction of the paravertebral muscles and posterior spinal ligament complex during lumbar fusion surgery is also an important cause of postoperative degeneration of adjacent segments. Among the paravertebral muscle groups, the superficial large muscle groups are mainly responsible for counteracting extrinsic loads and maintaining the posture of the whole spine. The deep intersegmental muscle groups are mainly responsible for maintaining the stability of the lumbar spine [50]. When the paravertebral muscles and soft tissues are damaged too much to perform normal physiological functions, it will lead to loss of spinal balance and compensatory biomechanical changes in the triple joint complex. When the loss of balance exceeds the compensatory capacity of the triple joint complex, degenerative changes in the triple joint complex will be accelerated.

**Fig. 7:**
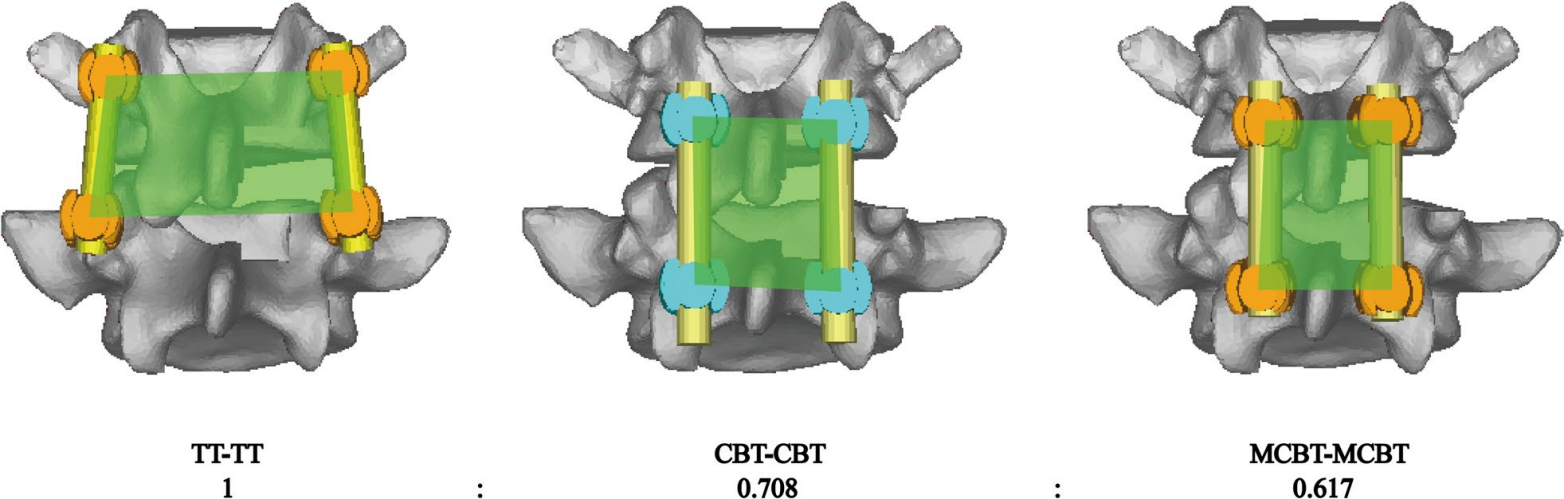
Proportion of surgically exposed area in three fixation techniques

Fusion surgery often requires blunt separation of the paravertebral muscles and partial resection of the facet joints to expose the surgical field of view, including TLIF. This process causes some damage to the paravertebral muscles and soft tissues, further aggravating the degeneration of the triple joint complex. A smaller intraoperative invasion of the deep muscle groups of the multifidus, soft tissues, blood vessels, and nerves can not only reduce postoperative pain and infection but also decrease the length of hospital stay [51], which is especially important for obese patients. In this experiment, we calculated and compared the corresponding values on the finite element model for the range and proportion of muscle area that needs to be stripped for the three internal fixation techniques (Fig. 7). Among them, the TT technique has the largest surgical exposure area, and the implantation of TT screws requires extensive stripping and pulling of soft tissues such as fascia, muscles, and ligaments, which causes severe damage to the soft tissues of the patient's surgical site, which not only affects the stress of the adjacent segments but also affects the fixed segment itself and even leads to loosening of the internal fixation [52]. The second is the surgical exposure area of the CBT technique. and the CBT technique reduces the incidence of ASD by shifting the screw placement point internally and changing the angle of the screw path, which avoids extensive exposure of the articular eminence joint to a certain extent and reduces muscle stripping and surgical trauma [49]. The technique with minimal posterior surgical exposure is the MCBT technique, which differs from the CBT technique mainly in that the screw placement point is closer to the midline of the spinous process in the longitudinal axis and has a greater abduction angle. This advantage minimizes surgical field exposure, and muscle stripping, and protects the nerve roots, the dural sac, and large blood vessels in the anterior spine during screw placement [11, 12]. However, the MCBT screws have some disadvantages in the case of small vertebral bodies for that screw placement is easily affected by the spinous process, requiring thinning of the spinous process or removal of a portion of the spinous process. In patients with lateral saphenous fossa stenosis, decompression may be limited due to concerns about the position of the screw placement point being affected, and neurologic symptoms may be incompletely relieved. Additionally, the MCBT technique is only possible in the lumbar 3, 4, and 5 vertebrae, which have a large transverse pedicle diameter, but not in the lumbar 1 and 2 vertebrae, which have a small transverse pedicle diameter. The MCBT technique is more suitable for patients with central canal stenosis, and its applicability can be further increased by combining it with spinal endoscopic technique [11–13].

### Limitations of this study

While finite element models offer valuable insights, they are simplifications with inherent limitations compared to real-world clinical scenarios. First, models rely on assumptions and approximations that may not fully capture the intricacies of biomechanics and physiology. Small errors could compound. Second, replicating the variability amongst patients seen in daily practice poses challenges. Factors like age, gender, BMI, bone density, etc. impact outcomes. Third, models are restricted by input accuracy. Using imprecise material properties or loading conditions reduces applicability. Fourth, capturing complex biological responses like bone remodeling over time is difficult, necessitating long-term clinical data. Fifth, models estimate trends but cannot precisely predict individual patient outcomes or substitute clinical judgment of risks versus benefits.

To further improve the reliability of the results of spinal biomechanical finite element analysis, the following aspects can be considered as follows: Accuracy of the material model, accuracy of geometric modeling, reasonable mesh delineation, accuracy of boundary conditions, sensitivity analysis, and result verification. To translate these findings into practice, clinical validation is essential. Outcomes predicted by models should be compared to observations from physical experiments and patient studies, with discrepancies noted and used to refine the models. Additionally, FE models likely function optimally complementing rather than replacing physician expertise, supporting rather than supplanting clinical decision-making.

## Conclusions

In conclusion, after fixation of the diseased L4-L5 segments using the three fixation techniques in TLIF, the MCBT-MCBT group was able to better equalize the ROM of the upper and lower adjacent segments in almost all motions compared to the CBT-CBT and TT-TT groups, with keeping the center of spinal motion in the fused segment and the lower lumbar spine. And it will conduce to protecting the degeneration-prone superior adjacent segments. However, the MCBT-MCBT group showed higher peak von Mises stress of the disc in the L5-S1 segment in almost all motions than the CBT-CBT group, so the protective effect of the L5-S1 segment disc may not be strong. But the screw placement point of the MCBT technique is more distant from the articular facet joint and the surgical exposure area is smaller, which maximizes the protection of the articular facet joint and muscle soft tissue damage. Therefore, we believe that the MCBT technique has some advantages in reducing the incidence of ASD after lumbar fusion.

## Data Availability

The raw data supporting the conclusion of this article will be made available by the Corresponding author, without undue reservation.

## Abbreviations

TLIF: Transforaminal lumbar interbody fusion
PLIF: Posterior Lumbar Interbody Fusion
TT: Traditional trajectory
CBT: Cortical bone trajectory
MCBT: Modified cortical bone trajectory
FE: Finite element
ROM: Range of motion

## References

[1] Anderson CE (1956). Spondyloschisis following spine fusion. J Bone Joint Surg Am.

[2] Park P, Garton HJ, Gala VC, Hoff JT, McGillicuddy JE (2004). Adjacent segment disease after lumbar or lumbosacral fusion: a review of the literature. Spine (Phila Pa 1976).

[3] Harms J, Rolinger H (1982). Die operative Behandlung der Spondylolisthese durch dorsale Aufrichtung und ventrale Verblockung [A one-stager procedure in operative treatment of spondylolistheses: dorsal traction-reposition and anterior fusion (author's transl)]. Z Orthop Ihre Grenzgeb.

[4] Fleege C, Rickert M, Rauschmann M (2015). PLIF- und TLIF-Verfahren. Indikation, Technik, Vor- und Nachteile [The PLIF and TLIF techniques. Indication, technique, advantages, and disadvantages]. Orthopade.

[5] Verma K, Boniello A, Rihn J (2016). Emerging Techniques for Posterior Fixation of the Lumbar Spine. J Am Acad Orthop Surg.

[6] Hollensteiner M, Sandriesser S, Bliven E, von Rüden C, Augat P (2019). Biomechanics of Osteoporotic Fracture Fixation. Curr Osteoporos Rep.

[7] Ding H, Hai Y, Liu Y, et al. Cortical Trajectory Fixation Versus Traditional Pedicle-Screw Fixation in the Treatment of Lumbar Degenerative Patients with Osteoporosis: A Prospective Randomized Controlled Trial. Clin Interv Aging. 2022;17:175–184. Published 2022 Feb 23. 10.2147/CIA.S349533 10.2147/CIA.S349533 PMC8882472 35237030

[8] Umehara S, Zindrick MR, Patwardhan AG (2000). The biomechanical effect of postoperative hypolordosis in instrumented lumbar fusion on instrumented and adjacent spinal segments. Spine (Phila Pa 1976).

[9] Santoni BG, Hynes RA, McGilvray KC (2009). Cortical bone trajectory for lumbar pedicle screws. Spine J.

[10] Matsukawa K, Yato Y, Kato T, Imabayashi H, Asazuma T, Nemoto K (2014). In vivo analysis of insertional torque during pedicle screwing using cortical bone trajectory technique. Spine (Phila Pa 1976).

[11] Rexiti P, Abudurexiti T, Abuduwali N, Wang S, Sheng W. Measurement of lumbar isthmus parameters for novel starting points for cortical bone trajectory screws using computed radiography. Am J Transl Res. 2018;10(8):2413–2423. Published 2018 Aug 15. PMC6129534 30210680

[12] Rexiti P, Aierken G, Wang S, et al. Anatomical research on strength of screw track fixation in novel cortical bone trajectory for osteoporosis lumbar spine. Am J Transl Res. 2019;11(11):6850–6859. Published 2019 Nov 15. PMC6895526 31814892

[13] Rexiti P, Aierken A, Sadeer A (2020). Anatomy and Imaging Studies on Cortical Bone Screw Freehand Placement Applying Anatomical Targeting Technology. Orthop Surg.

[14] Fujiwara S, Ohnishi Y, Iwatsuki K, Kishima H. Cortical bone trajectory fixation cause low compression force in anterior vertebral column. N Am Spine Soc J. 2022;10:100113. Published 2022 Mar 18. 10.1016/j.xnsj.2022.100113 10.1016/j.xnsj.2022.100113 PMC9005947 35434674

[15] Maitirouzi J, Luo H, Zhou Z, Ren H, Rexiti P (2022). Finite element analysis of human lumbar vertebrae in internal fixation system model with different bone density trajectories. Int J Artif Organs.

[16] Zhang R, Kahaer A, Niu H, et al. Biomechanical evaluation of the hybrid pedicle screw-cortical bone trajectory technique in transforaminal lumbar interbody fusion to adjacent segment degeneration-finite element analysis [published correction appears in BMC Musculoskelet Disord. 2023 Jul 5;24(1):555]. BMC Musculoskelet Disord. 2023;24(1):409. Published 2023 May 23. 10.1186/s12891-023-06411-z 10.1186/s12891-023-06411-z PMC10204234 37221546

[17] Zander T, Rohlmann A, Bergmann G (2009). Influence of different artificial disc kinematics on spine biomechanics. Clin Biomech (Bristol, Avon).

[18] Xu H, Ju W, Xu N (2013). Biomechanical comparison of transforaminal lumbar interbody fusion with 1 or 2 cages by finite-element analysis. Neurosurgery.

[19] Wang MdK, Jiang PhDC, Wang PhDL (2018). The biomechanical influence of anterior vertebral body osteophytes on the lumbar spine: A finite element study. Spine J.

[20] Kahaer A, Zhang R, Wang Y, et al. Hybrid pedicle screw and modified cortical bone trajectory technique in transforaminal lumbar interbody fusion at L4-L5 segment: finite element analysis. BMC Musculoskelet Disord. 2023;24(1):288. Published 2023 Apr 13. 10.1186/s12891-023-06385-y 10.1186/s12891-023-06385-y PMC10099636 37055739

[21] Matsukawa K, Yato Y, Nemoto O, Imabayashi H, Asazuma T, Nemoto K (2013). Morphometric measurement of cortical bone trajectory for lumbar pedicle screw insertion using computed tomography. J Spinal Disord Tech.

[22] Sansur CA, Caffes NM, Ibrahimi DM (2016). Biomechanical fixation properties of cortical versus transpedicular screws in the osteoporotic lumbar spine: an in vitro human cadaveric model. J Neurosurg Spine.

[23] Matsukawa K, Yato Y, Imabayashi H, Hosogane N, Abe Y, Asazuma T, Chiba K (2016). Biomechanical evaluation of fixation strength among different sizes of pedicle screws using the cortical bone trajectory: what is the ideal screw size for optimal fixation?. Acta Neurochir (Wien).

[24] Chen WJ, Wang HL, Jiang JY (2015). Anatomic study on lumbar cortical bone trajectory of adults. Chin J Orthop.

[25] Zhou ZH. Biomechanical properties of modified cortical bone trajectory technique: a finite element analysis. Xinjiang Medical Universit.2022.

[26] Yamamoto I, Panjabi MM, Crisco T, Oxland T (1989). Three-dimensional movements of the whole lumbar spine and lumbosacral joint. Spine.

[27] Shim CS, Park SW, Lee SH, Lim TJ, Chun K, Kim DH (2008). Biomechanical evaluation of an interspinous stabilizing device. Locker Spine.

[28] Huang YP, Du CF, Cheng CK, Zhong ZC, Chen XW, Wu G (2016). Preserving posterior complex can prevent adjacent segment disease following posterior lumbar interbody fusion surgeries: a finite element analysis. PLoS ONE.

[29] Lo HJ, Chen HM, Kuo YJ, Yang SW (2020). Effect of different designs of interspinous process devices on the instrumented and adjacent levels after double-level lumbar decompression surgery: A finite element analysis. PLoS ONE.

[30] Stoffel M, Behr M, Reinke A, Stüer C, Ringel F, Meyer B (2010). Pedicle screw-based dynamic stabilization of the thoracolumbar spine with the Cosmic-system: a prospective observation. Acta Neurochir (Wien).

[31] Sears WR, Sergides IG, Kazemi N, Smith M, White GJ, Osburg B (2011). Incidence and prevalence of surgery at segments adjacent to a previous posterior lumbar arthrodesis. Spine J.

[32] Lee JC, Kim Y, Soh JW, Shin BJ (2014). Risk factors of adjacent segment disease requiring surgery after lumbar spinal fusion: comparison of posterior lumbar interbody fusion and posterolateral fusion. Spine (Phila Pa 1976).

[33] Kumar MN, Baklanov A, Chopin D (2001). Correlation between sagittal plane changes and adjacent segment degeneration following lumbar spine fusion. Eur Spine J.

[34] Lee CK, Langrana NA (1984). Lumbosacral spinal fusion. A biomechanical study. Spine (Phila Pa 1976).

[35] Willburger RE, Krämer J, Wiese M (2005). Chirurgische Anatomie der Lendenwirbelsäule [Surgical anatomy of the lumbar spine]. Orthopade.

[36] Pope MH (1989). Biomechanics of the lumbar spine. Ann Med.

[37] Zhang L (2021). Effect of cortical bone trajectory screw on inserted and adjacent segments in lumber vertebra: A biomechanics study by FEM. Anhui Medical University.

[38] Matsukawa K, Yato Y, Imabayashi H, Hosogane N, Asazuma T, Nemoto K (2015). Biomechanical evaluation of the fixation strength of lumbar pedicle screws using cortical bone trajectory: a finite element study. J Neurosurg Spine.

[39] Claeson AA, Barocas VH (2017). Computer simulation of lumbar flexion shows shear of the facet capsular ligament. Spine J.

[40] Vanharanta H, Floyd T, Ohnmeiss DD, Hochschuler SH, Guyer RD (1993). The relationship of facet tropism to degenerative disc disease. Spine (Phila Pa 1976).

[41] Fei Q, Lin JS, WANG BQ, Yang Y, Li D, MA Z, Zhao F, WANG Q (2014). Research progress of the role of facet joints in lumbar instability. J Clin Exp.

[42] Patel JY, Kundnani VG, Merchant ZI, Jain S, Kire N (2020). Superior Facet Joint Violations in Single Level Minimally Invasive and Open Transforaminal Lumbar Interbody Fusion: A Comparative Study. Asian Spine J.

[43] Zeng ZL, Jia L, Xu W, et al. Analysis of risk factors for adjacent superior vertebral pedicle-induced facet joint violation during the minimally invasive surgery transforaminal lumbar interbody fusion: a retrospective study. Eur J Med Res. 2015;20:80. Published 2015 Sep 24. 10.1186/s40001-015-0174-9 10.1186/s40001-015-0174-9 PMC4581410 26399320

[44] Fu L, Ma JX, Ma XL (2015). Research progress on biomechanics of facet joints. Chin J Orthop.

[45] Song X, Cao SF, Ren DL (2017). Incidence and risk factors of adjacent cranial facet joint violation following pedicle screw insertion using Weinstein technique in TLIF. J Tongji Univ.

[46] Chung KJ, Suh SW, Swapnil K, Yang JH, Song HR (2007). Facet joint violation during pedicle screw insertion: a cadaveric study of the adult lumbosacral spine comparing the two pedicle screw insertion techniques. Int Orthop.

[47] He B, Yan L, Guo H, Liu T, Wang X, Hao D (2014). The difference in superior adjacent segment pathology after lumbar posterolateral fusion by using 2 different pedicle screw insertion techniques in 9-year minimum follow-up. Spine (Phila Pa 1976).

[48] Zhang Q, Han XG, Xu YF (2019). Robot-Assisted Versus Fluoroscopy-Guided Pedicle Screw Placement in Transforaminal Lumbar Interbody Fusion for Lumbar Degenerative Disease. World Neurosurg.

[49] Mobbs RJ. The “medio-latero-superior trajectory technique”: an alternative cortical trajectory for pedicle fixation. Orthop Surg. 2013;5(1):56–9. 10.1111/os.12027. 10.1111/os.12027 PMC6583172 23420749

[50] Hu Y. Electrophysiological and PerformanceChanges of the Lumbar Paraspinal Muscle After Posterior Lumbar Interbody Fusion. Chinese PLA Medical College.2012

[51] Calvert GC, Lawrence BD, Abtahi AM, Bachus KN, Brodke DS (2015). Cortical screws used to rescue failed lumbar pedicle screw construct: a biomechanical analysis. J Neurosurg Spine.

[52] Kim JB, Park SW, Lee YS, Nam TK, Park YS, Kim YB (2015). The Effects of Spinopelvic Parameters and Paraspinal Muscle Degeneration on S1 Screw Loosening. J Korean Neurosurg Soc.

